# Plastid Phylogenomics of Elatostemateae (Urticaceae): Insights Into Structural Variation and Phylogenetic Relationships

**DOI:** 10.1002/ece3.74416

**Published:** 2026-10-06

**Authors:** Long‐Fei Fu, Zi‐Bing Xin, Chi Xiong, Fang Wen, Yi‐Gang Wei, Alexandre K. Monro

**Affiliations:** ^1^ Guangxi Zhuang Autonomous Region and Chinese Academy of Sciences Guangxi Key Laboratory of Plant Conservation and Restoration Ecology in Karst Terrain, Guangxi Institute of Botany Guilin China; ^2^ Identification & Naming Department The Royal Botanic Gardens, Kew Richmond UK

**Keywords:** *Elatostema*, *Gyrotaenia*, phylogenomics, *Pilea*, plastome structural variation, systematics, Urticaceae

## Abstract

The tribe Elatostemateae, with approximately 1300 species, is the most species‐rich group within Urticaceae, yet plastome evolution and several intergeneric relationships remain incompletely resolved. We analyzed 40 species representing the major extant lineages of Elatostemateae using complete plastomes and nuclear ribosomal DNA (nrDNA); *Metapilea* was excluded because suitable DNA material was unavailable. Plastomes retained the typical angiosperm quadripartite structure and ranged from 143,738 to 163,969 bp. Comparative analyses revealed large inversions in the large single‐copy (LSC) region, pronounced inverted‐repeat (IR) boundary shifts, and a rare plastid mitochondrial DNA‐derived sequence (PTMT) insertion. Seven highly variable regions were identified as candidate markers for DNA barcoding. Plastome, protein‐coding sequence (CDS), nrDNA, and combined analyses recovered the same three major Elatostemateae clades, although several internal nrDNA nodes had lower support. Plastid and nrDNA data placed *Gyrotaenia* as sister to *Pilea s.l*. within Elatostemateae, whereas recent low‐copy nuclear evidence places *Gyrotaenia* + *Myriocarpa* sister to core Elatostemateae, revealing cytonuclear discordance. These results provide a framework for Elatostemateae systematics and document substantial plastome structural variation; denser low‐copy nuclear or whole‐genome sampling will be needed to distinguish incomplete lineage sorting from historical introgression.

## Graphical abstract

This study leverages complete plastid and nuclear ribosomal DNA sequences from 40 species to resolve the complex phylogeny and organellar genome evolution of the diverse plant tribe Elatostemateae. The analysis uncovered significant plastome structural variations, including novel inversions and a rare mitochondrial DNA integration event, and identified seven hypervariable regions for future DNA barcoding. Ultimately, these multi‐locus genomic data provide a robust evolutionary framework, confirming the monophyly of the tribe and its genera while revealing the unexpected nesting of Gyrotaenia within it due to potential ancestral introgression.

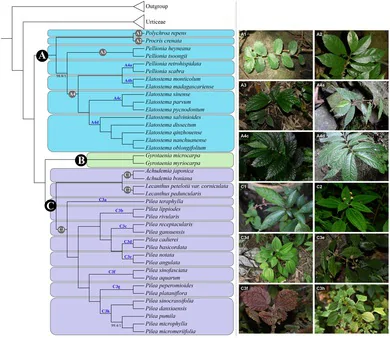

## 1 Introduction

The tribe Elatostemateae Gaudich. (Urticaceae), a pantropical group encompassing approximately 1300 species, represents the most species‐rich tribe within the family (Friis 1989; Wu et al. 2013). Its diversity spans delicate herbs to woody shrubs, and members commonly occupy shaded, humid habitats including forest understories, stream sides, limestone karst gorges, and cave systems (Monro et al. 2018; Wu et al. 2018; Tseng et al. 2019; Fu et al. 2022). Elatostemateae is differentiated from allied tribes by a combination of reproductive traits, particularly penicillate stigmas and a specialized achene‐ejection mechanism mediated by staminodes (Friis 1989). At the generic level, classical circumscriptions additionally use combinations of leaf arrangement and symmetry, stipule position, inflorescence architecture, floral merosity, receptacle and bracteole development, and achene morphology. These characters remain useful for identification but can be homoplastic, which has motivated repeated molecular reassessment of generic boundaries (Monro 2006; Tseng et al. 2019; Fu et al. 2022; Monro et al. 2025).

Early phylogenetic reconstructions of Elatostemateae, largely reliant on Sanger sequencing of a limited gene set, often yielded poorly resolved or incongruent phylogenetic hypotheses. For example, global sampling initiatives designed to elucidate relationships within the broader Urticaceae framework validated the core monophyly of Elatostemateae, yet simultaneously revealed the polyphyletic nature of the genus *Pellionia*, prompting proposals to incorporate genera such as *Sarcopilea* directly within *Pilea* (Wu et al. 2013; Monro 2006). Furthermore, broad‐scale taxonomic sampling of the allied tribe Urticeae confirmed its respective monophyly while identifying the genus *Gyrotaenia* as the putative sister group to Elatostemateae (Kim et al. 2015). Advancing these inquiries, subsequent studies utilizing target enrichment phylogenomics combined with restriction‐site data recovered a divergent clade comprising *Gyrotaenia* and *Myriocarpa* positioned as sister to the core Elatostemateae (Monro et al. 2025).

Substantial revisions have also continuously refined relationships at the generic level. The monophyly of *Pilea* was affirmed through targeted sampling encompassing major morphological, taxonomic, and biogeographic groups, establishing a correlation between phylogenetic lineage divergence and global geographic distribution (Monro 2006). Following the discovery of *Metapilea* W.T.Wang as a new genus (Wang 2016), extensive molecular evaluations of *Elatostema sensu lato (s.l.)* revised the strict circumscription of *Elatostema* (by excluding *Pellionia repens* ≡ *Polychroa repens*) and subsequently elevated *Elatostematoides* and *Procris* to the status of distinct genera (Tseng et al. 2019). More recently, deep molecular sampling within *Pilea s.l*. further reshaped generic boundaries, establishing *Achudemia* and *Lecanthus* as separate monophyletic entities while advancing a modernized infrageneric classification for *Pilea* (Fu et al. 2022). Based on these cumulative systematic revisions, eight genera are recognized in the sampling framework used here: *Achudemia*, *Elatostema*, *Elatostematoides*, *Lecanthus*, *Metapilea*, *Pilea*, *Polychroa*, and *Procris*.

Despite the progressive clarification of species relationships, the comprehensive study of plastome evolution within Elatostemateae remains largely absent from the literature, representing a bottleneck in understanding the tribe's deeper evolutionary history. Driven by its relatively conserved structural architecture, maternal inheritance (in most angiosperms), and high accessibility via next‐generation sequencing paradigms, the plastome functions as a useful instrument for dissecting complex evolutionary trajectories (Twyford and Ness 2017; Guo et al. 2023; Wang et al. 2024). Comparative plastome analyses consistently offer resolution in delineating phylogenetic nodes ranging from shallow species complexes to deep tribal divergences. Moreover, such analyses yield crucial data concerning macro‐genomic evolution, uncovering mechanisms driving structural rearrangements, inversions, and large‐scale variations in genetic content (Villarreal et al. 2013; Dugas et al. 2015; Choi and Choi 2017; Sinn et al. 2018; Wu et al. 2024).

While comparative plastome investigations within Urticaceae have produced insights into the tribes Boehmerieae and Urticeae (uncovering extensive structural variations that redefine their phylogenies) (Wang et al. 2020; Ogoma et al. 2022; Wu, Milne, et al. 2022; Zhan et al. 2024), the specific plastome dynamics within Elatostemateae have been largely ignored. Previous genomic surveys within this tribe have been restricted to isolated descriptions of single species or highly localized generic groupings (Fu et al. 2021; Li et al. 2021; Tseng et al. 2025). This fragmented approach restricts holistic phylogenetic inferences and obscures broader patterns of plastid genome evolution.

To address unresolved intergeneric relationships and the lack of a comparative plastome framework for Elatostemateae, this study integrates whole‐plastome architecture with nuclear ribosomal DNA (nrDNA) sequence data. The objectives were to (1) improve resolution of phylogenetic relationships among genera and major morphological clades within the tribe; (2) characterize plastome structural variation, including inverted‐repeat boundary shifts and rearrangements, and assess its phylogenetic and taxonomic significance; and (3) identify candidate hypervariable chloroplast DNA (cpDNA) regions for future species identification and DNA barcoding.

## 2 Materials and Methods

### 2.1 Taxon Sampling, DNA Extraction, and Sequencing

We sampled 40 species (40 accessions) representing the extant genera included in our Elatostemateae circumscription, excluding the monotypic *Metapilea*, which is likely extinct and for which suitable DNA material was unavailable (Monro et al. 2025). Sampling covered major morphological clades and recognized sections within the two most diverse and taxonomically complex genera, *Elatostema* and *Pilea*. Species identifications were verified from voucher specimens and current taxonomic treatments; voucher, locality, herbarium, and accession information is provided in Table 1.

**TABLE 1:** Sampling information and Genbank accession numbers of plastomes and nrDNA used in this study.

Species	Voucher	Country	Herbarium	Genbank accession		Tribe	Category
				Plastome	nrDNA		
*Achudemia boniana*	FLF2001	Longzhou, China	IBK	PV715426*	PV642555*	Elatostemateae	Ingroup
*Achudemia japonica*	DJ20171123085	Guangxi, China	IBK	PV715427*	PV642558*	Elatostemateae	Ingroup
*Elatostema dissectum*	XZB20180131‐01	Guangxi, China	IBK	NC 047192	PV642541*	Elatostemateae	Ingroup
*Elatostema madagascariense*	2105A	Africa	BM	PV715419*	PV642542*	Elatostemateae	Ingroup
*Elatostema monticolum*	MW0586079	Ethiopia	MW	PV715420*	PV642543*	Elatostemateae	Ingroup
*Elatostema nanchuanense*	WF180109‐05	Yunnan, China	IBK	PV715440*	PV642544*	Elatostemateae	Ingroup
*Elatostema oblongifolium*	XZB20180116‐11	Guizhou, China	IBK	PV715436*	PV642545*	Elatostemateae	Ingroup
*Elatostema parvum*	FL0046	Yunnan, China	IBK	PV715441*	PV642546*	Elatostemateae	Ingroup
*Elatostema pycnodontum*	DJ2018011620	Guizhou, China	IBK	PV715439*	PV642575*	Elatostemateae	Ingroup
*Elatostema qinzhouense*	P1184	Guangxi, China	IBK	MW172521	MW172522	Elatostemateae	Ingroup
*Elatostema sinense*	DJ20180116‐116	Guizhou, China	IBK	PV715442*	PV642547*	Elatostemateae	Ingroup
*Elatostema salvinioides*	WF180122‐03	Yunnan, China	IBK	NC 069593	PV698328*	Elatostemateae	Ingroup
*Lecanthus peduncularis*	WF180817‐05	Xizang, China	IBK	PV715424*	PV642548*	Elatostemateae	Ingroup
*Lecanthus petelotii var. corniculata*	WF200819	Xizang, China	IBK	PV715425*	PV642574*	Elatostemateae	Ingroup
*Pellionia heyneana*	KL 32900	Tailand	K	PV715422*	PV642549*	Elatostemateae	Ingroup
*Pellionia repens*	VMN‐CN1159	Vietnam	IBK	PV715423*	PV642550*	Elatostemateae	Ingroup
*Pellionia retrohispidata*	XC22050	Chongqing, China	IBK	OR521094*	PV642573*	Elatostemateae	Ingroup
*Pellionia scabra*	XC22051	Chongqing, China	IBK	OR521097*	PV642572*	Elatostemateae	Ingroup
*Pellionia tsoongii*	WYG180319‐04	Vietnam	IBK	PV715421*	PV642551*	Elatostemateae	Ingroup
*Pilea angulata*	WFLSH120819	Guangxi, China	IBK	PV715447*	PV642552*	Elatostemateae	Ingroup
*Pilea aquarum*	Wei097	Guangxi, China	IBK	PV715435*	PV642553*	Elatostemateae	Ingroup
*Pilea basicordata*	AM6517	Guangxi, China	IBK	PV715434*	PV642554*	Elatostemateae	Ingroup
*Pilea cadierei*	HSL098	Guangxi, China	IBK	PV715446*	PV642556*	Elatostemateae	Ingroup
*Pilea danxiaensis*	17556	Guangdong, China	SYS	ON496932	ON557625	Elatostemateae	Ingroup
*Pilea gansuensis*	870233	Sichuan, China	LE	PV715431*	PV642557*	Elatostemateae	Ingroup
*Pilea lippiodes*	15559	Africa	LE	PV715430*	PV642559*	Elatostemateae	Ingroup
*Pilea micromeriifolia*	MW0573763	Cuba	MW	PV715438*	PV642561*	Elatostemateae	Ingroup
*Pilea microphylla*	HSL024	Yunnan, China	IBK	PV715445*	PV642560*	Elatostemateae	Ingroup
*Pilea notata*	WFLSH120925	Guangdong, China	IBK	PV715433*	PV642562*	Elatostemateae	Ingroup
*Pilea plataniflora*	HSL018	Yunnan, China	IBK	PV715444*	PV642564*	Elatostemateae	Ingroup
*Pilea pumila*	MW0062592	Russia	MW	PV715443*	PV642565*	Elatostemateae	Ingroup
*Pilea receptacularis*	ZXX001	Sichuan, China	IBK	PV715432*	PV642566*	Elatostemateae	Ingroup
*Pilea rivularis*	MW0573762	Cuba	MW	PV715429*	PV642567*	Elatostemateae	Ingroup
*Pilea sinocrassifolia*	FLF190801‐02	Guangxi, China	IBK	NC 069841	PV642568*	Elatostemateae	Ingroup
*Pilea sinofasciata*	ZXX002	Sichuan, China	IBK	PV715437*	PV642569*	Elatostemateae	Ingroup
*Pilea teraphylla*	MW0586078	Ethiopia	MW	PV715428*	PV642570*	Elatostemateae	Ingroup
*Pilea peperomioides*	—	Guizhou, China	IBK	PV715448*	PV642563*	Elatostemateae	Ingroup
*Procris crenata*	DJ20170922pr	Guangxi, China	IBK	NC 056276	PV642571*	Elatostemateae	Ingroup
*Gyrotaenia microcarpa*	68859	Mexico	K	OM761911	OM892727	Myriocarpeae	Ingroup
*Gyrotaenia myriocarpa*	7479	Dominican	K	OM761912	OM892726	Myriocarpeae	Ingroup
*Broussonetia monoica*	—	—	—	—	MH135780	—	Outgroup
*Broussonetia papyrifera*	—	—	—	—	MH135784	—	Outgroup
*Ficus virens*	—	—	—	MW039142	—	—	Outgroup
*Maclura tricuspidata*	—	—	—	MW244565	—	—	Outgroup
*Boehmeria tomentosa*	WuZY‐09011	China	KUN	MN189945	OM892757	Boehmerieae	Outgroup
*Debregeasia elliptica*	WuZY‐10134	China	KUN	MN189948	OM892755	Boehmerieae	Outgroup
*Oreocnide frutescens*	—	—	—	MT465830	—	Boehmerieae	Outgroup
*Rousselia humilis*	8274	Dominican Republic	K	OM761933	OM892777	Boehmerieae	Outgroup
*Cecropia pachystachya*	Bruno5‐2017	Brazil	KUN	OM761898	OM892756	Cecropieae	Outgroup
*Forsskaolea angustifolia*	2878	Planted in Kew		MT465767	OM892746	Parietarieae	Outgroup
*Parietaria floridana*	—	—	—	MT465764	—	Parietarieae	Outgroup
*Parietaria micrantha*	WuZY‐10373	China	KUN	MN189966	OM892770	Parietarieae	Outgroup
*Dendrocnide basirotunda*	12CS5733	China	KUN	OM761900	OM892754	Urticeae	Outgroup
*Dendrocnide sinuata*	13CS6832	China	KUN	OM761903	OM892751	Urticeae	Outgroup
*Giradinia bullosa*	KE002	Kenya	KUN	OM877286	OM892745	Urticeae	Outgroup
*Girardinia chingiana*	liuj‐10717	China	KUN	OM877274	OM892742	Urticeae	Outgroup
*Girardinia diversifolia*	RC1817	Nepal	KUN	OM877271	OM892738	Urticeae	Outgroup
*Hesperocnide tenella*	USA	E00850023	E	OM761913	OM892725	Urticeae	Outgroup
*Laportea bulbifera*	GLGE14842	China	KUN	OM761918	OM892720	Urticeae	Outgroup
*Laportea cuspidata*	TianXH263	China	K	OM761921	OM892717	Urticeae	Outgroup
*Laportea decumana*	C8746	Indonesia	K	OM877281	OM892816	Urticeae	Outgroup
*Laportea grossa*	WH01	South Africa	KUN	OM877280	OM892759	Urticeae	Outgroup
*Laportea medogensis*	GLGE141037	China	KUN	OM877279	OM892760	Urticeae	Outgroup
*Laportea mooreana*	14811	—	K	OM761923	OM892761	Urticeae	Outgroup
*Laportea ovalifolia*	951	Nigeria	K	OM761924	OM892762	Urticeae	Outgroup
*Nanocnide japonica*	liuj‐10746	China	KUN	OM761926	OM892764	Urticeae	Outgroup
*Nanocnide lobata*	GanQL278	China	KUN	OM761927	OM892765	Urticeae	Outgroup
*Obetia aldabrensis*	1358	Seychelles	K	OM761929	OM892767	Urticeae	Outgroup
*Obetia radula*	326	Tanzania	K	OM877290	OM892768	Urticeae	Outgroup
*Poikilospermum cordifolium*	CWL.1	Singapore	K	OM761930	OM892772	Urticeae	Outgroup
*Poikilospermum lanceolatum*	S4494	China	KUN	OM761932	OM892774	Urticeae	Outgroup
*Urera baccifera*	9103	Bolivia	K	OM761935	OM892779	Urticeae	Outgroup
*Urera capitata*	7608	Bolivia	K	OM877277	OM892782	Urticeae	Outgroup
*Urera hypselodendron*	IM86	Sudan	K	OM761937	OM892785	Urticeae	Outgroup
*Urera robusta*	5398	Colony Province, Gold coast	K	OM761938	OM892787	Urticeae	Outgroup
*Urtica dioica*	FLAS219395	USA	FLAS	OM761947	OM892796	Urticeae	Outgroup
*Urtica mairei*	12CS4575	China	KUN	OM761952	OM892802	Urticeae	Outgroup
*Zhengyia shennongensis*	Liuj14656	China	KUN	OM761908	OM892811	Urticeae	Outgroup

*Note:* Asterisks (*) denote newly generated sequences.

Total genomic DNA was isolated from silica‐dried leaf tissue using a modified cetyltrimethylammonium bromide (CTAB) protocol (Chen et al. 2014), and DNA integrity was assessed using 1% agarose gel electrophoresis. Following quality control, library construction and sequencing were performed by Majorbio Bio‐pharm Technology Co. Ltd. (Shanghai, China) using the Illumina TruSeq DNA Library Preparation Kit. Libraries were sequenced on an Illumina HiSeq 4000 platform to generate 2 × 150‐bp paired‐end reads, with approximately 2 Gb of genomic data per sample. Raw reads were processed with FASTX‐Toolkit to remove adapter‐contaminated and low‐quality reads, applying specific numerical filtering thresholds to discard bases with a quality score < 20, reads shorter than 35 bp, and sequences containing homopolymers > 6 bases; all downstream analyses used the resulting clean reads.

### 2.2 Assembly and Computational Annotation

Filtered reads were assembled de novo with GetOrganelle v1.7.6.1 (Jin et al. 2020); no reference plastome was used. For plastome assembly, k‐mer sizes spanning 65–121 bp were explored, with other settings left at program defaults. The corresponding nrDNA region was recovered from the same genome‐skimming reads using the de novo GetOrganelle framework specifically utilizing the embryophyte nrDNA database as the seed, with k‐mer sizes of 35, 85, and 115 bp, and maximum extension rounds set to 10. The resulting assemblies were then checked for the expected 18S‐ITS1‐5.8S‐ITS2‐26S organization. Complete plastomes were annotated independently with PGA and CPGAVAS2 to verify gene boundaries (Qu et al. 2019; Shi et al. 2019). Circular plastome maps were generated with OGDRAW v1.3.1 (Greiner et al. 2019).

### 2.3 Phylogenetic Reconstruction

Phylogenetic analyses included 74 complete plastomes and 74 corresponding nrDNA sequences. The matrix comprised the 40 Elatostemateae ingroup taxa together with representatives of other Urticaceae lineages and four Moraceae species used to root the trees: *Broussonetia monoica* Hance, *Broussonetia papyrifera* (L.) L'Hér. ex Vent., *Ficus virens* Aiton, and *Maclura tricuspidata* Carrière. The broader outgroup sampling included representatives of Boehmerieae and Urticeae because of their established phylogenetic proximity to Elatostemateae (Wu et al. 2013). Accession numbers are provided in Table S1.

Four data partitions were analyzed: (a) complete plastomes (CP), after removing one inverted‐repeat copy and realigning locally inverted regions before the final alignment to avoid duplicated or misaligned signal; (b) plastid protein‐coding sequences (CDS); (c) the nrDNA region spanning 18S‐ITS1‐5.8S‐ITS2‐26S; and (d) a concatenated CP + nrDNA dataset.

Phylogenetic relationships were inferred using Bayesian inference (BI) and maximum likelihood (ML). Sequences were aligned with MAFFT v7.407 using ‐‐auto and ‐‐adjustdirectionaccurately (Katoh and Standley 2013), and poorly aligned or gap‐rich columns were removed with trimAl v1.4.1 in automated mode (Capella‐Gutiérrez et al. 2009). Protein‐coding sequences were extracted from the annotated plastomes in Geneious Prime v9.0.2 and, after trimming, the individual CDS alignments were concatenated with the Concatenate Sequences or Alignments function (Kearse et al. 2012).

Best‐fitting nucleotide‐substitution models for BI and ML analyses were selected using jModelTest2 v2.1.7 and IQ‐TREE v1.6.12, respectively, under the Bayesian Information Criterion (BIC) (Darriba et al. 2012; Nguyen et al. 2015; Aho et al. 2014) (Table S1). BI analyses were conducted with MrBayes v3.2.7a through the CIPRES Science Gateway (Ronquist et al. 2012; Miller et al. 2011). Two independent runs of four Markov chain Monte Carlo chains were performed for 100 million generations, sampling every 1000 generations. The first 25% of sampled trees were discarded as burn‐in, and the remaining trees were summarized as a 50% majority‐rule consensus tree. Convergence between independent runs was assessed using the average standard deviation of split frequencies (ASDSF), with ASDSF ≤ 0.01 used as the convergence criterion. Effective sample sizes (ESS) > 200 for all monitored parameters were also required as an additional convergence criterion. ML analyses were conducted in IQ‐TREE v1.6.12 with 5000 standard nonparametric bootstrap replicates to estimate branch support. Phylogenetic trees were visualized with iTOL (Letunic and Bork 2021).

### 2.4 Plastome Structural Variation and Genome Architecture

Comparative analyses were performed across the 40 assembled Elatostemateae plastomes. Total plastome size, GC content, gene content, and the lengths of the large single‐copy (LSC), small single‐copy (SSC), and two inverted‐repeat regions (IRa and IRb) were summarized in Geneious. LSC/IRb (JLB), IRb/SSC (JSB), SSC/IRa (JSA), and IRa/LSC (JLA) junctions were defined from annotated region boundaries and visualized with CPJSDRAW (Li et al. 2023). Large‐scale rearrangements were assessed with Mauve using its default locally collinear block (LCB) search settings, with *Polychroa repens* as the structural reference (Darling et al. 2004).

### 2.5 Repeat Sequence Classification and Nucleotide‐Diversity Analysis

Repetitive sequences were classified into long repeats and simple sequence repeats (SSRs). Dispersed long repeats (forward, reverse, complement, and palindromic) were identified with REPuter using a Hamming distance of 3, a maximum of 50 repeats, and a minimum repeat length of 30 bp (Kurtz et al. 2001). Tandem long repeats were detected with Tandem Repeats Finder using default settings (Benson 1999).

Simple sequence repeats (SSRs) were identified with MISA using minimum repeat thresholds of 10, 5, 4, 3, 3, and 3 units for mono‐, di‐, tri‐, tetra‐, penta‐, and hexanucleotide motifs, respectively (Thiel et al. 2003). Nucleotide diversity (Pi) was calculated in DnaSP v6 (Rozas et al. 2017) for 100 genic regions and 95 intergenic spacers shared across the sampled plastomes. Sliding‐window analyses used a 600‐bp window and a 200‐bp step size.

## 3 Results

### 3.1 Plastome Organization and Characterization

Plastome size varied from 143,738 bp in *Pilea micromeriifolia* Britton & P.Wilson to 163,969 bp in *Elatostema oblongifolium* S.H.Fu (Table 2). All sampled plastomes retained the quadripartite structure common to photosynthetic angiosperms (Figure 1), comprising two IRs of 18,361–31,485 bp that separated an LSC region of 80,747–89,128 bp from an SSC region of 12,924–18,451 bp.

**TABLE 2:** Summary of features of the Elatostemateae plastomes.

Species	Whole size	LSC length	SSC length	IR length	GC%	LSC GC%	SSC GC%	IR GC%	Number of genes	Protein‐coding genes	tRNAs	rRNAs
*Pellionia repens*	154,704	85,126	18,354	25,612	36.56	34.16	30.26	42.81	129	84	37	8
*Procris crenata*	154,124	84,599	18,273	25,626	36.53	34.11	30.17	42.79	131	86	37	8
*Pellionia heyneana*	155,285	85,571	18,376	25,669	36.42	33.98	29.88	42.83	131	86	37	8
*Pellionia tsoongii*	154,518	84,842	18,310	25,683	36.51	34.09	29.97	42.83	131	86	37	8
*Pellionia retrohispidata*	153,563	84,825	17,638	25,550	36.43	33.92	29.82	42.87	131	86	37	8
*Pellionia scabra*	153,407	84,658	17,577	25,586	36.41	33.88	29.86	42.83	131	86	37	8
*Elatostema monticolum*	154,107	85,063	17,872	25,586	36.5	34.01	30.08	42.82	131	86	37	8
*Elatostema madagascariense*	154,050	85,014	17,864	25,586	36.48	34.02	30.05	42.81	131	86	37	8
*Elatostema sinense*	152,250	84,077	17,669	25,252	36.41	33.87	30.07	42.84	131	86	37	8
*Elatostema parvum*	152,343	84,012	17,753	25,289	36.36	33.82	29.84	42.86	131	86	37	8
*Elatostema pycnodontum*	152,508	84,295	17,633	25,290	36.36	33.82	29.91	42.85	131	86	37	8
*Elatostema salvinioides*	152,265	84,666	16,529	25,535	36.39	33.75	30.13	42.78	131	86	37	8
*Elatostema dissectum*	150,302	83,998	17,174	24,565	36.23	33.66	29.65	42.93	130	86	36	8
*Elatostema qinzhouense*	150,398	83,919	17,103	24,688	36.34	33.75	29.82	43	131	86	37	8
*Elatostema nanchuanense*	158,097	84,510	16,899	28,344	36.45	33.63	29.74	42.67	131	86	37	8
*Elatostema oblongifolium*	163,969	84,115	16,884	31,485	36.62	33.77	29.88	42.24	131	86	37	8
*Gyrotaenia microcarpa*	153,911	84,886	18,591	25,217	36.55	34.2	30.36	42.81	130	85	37	8
*Gyrotaenia myriocarpa*	153,979	84,963	18,596	25,210	36.53	34.16	30.27	42.81	130	85	37	8
*Achudemia japonica*	150,134	81,123	17,957	25,527	36.84	34.52	30.28	42.84	131	86	37	8
*Achudemia boniana*	149,560	80,747	17,797	25,508	36.87	34.52	30.25	42.9	130	85	37	8
*Lecanthus petelotii var. corniculata*	150,510	81,874	17,432	25,602	36.67	34.16	30.21	42.88	130	85	37	8
*Lecanthus peduncularis*	151,703	82,909	17,496	25,649	36.61	34.14	30.05	42.85	131	86	37	8
*Pilea teraphylla*	150,210	87,692	12,924	24,797	36.48	34.6	31.71	41.04	129	85	36	8
*Pilea lippiodes*	151,218	82,621	18,065	25,266	36.33	33.89	29.59	42.71	131	86	37	8
*Pilea rivularis*	151,222	81,658	18,006	25,779	36.42	34.03	29.69	42.56	132	87	37	8
*Pilea receptacularis*	152,330	82,748	18,030	25,776	36.5	34.11	29.94	42.62	132	87	37	8
*Pilea gansuensis*	152,041	82,460	18,019	25,781	36.51	34.13	29.92	42.62	132	87	37	8
*Pilea cadierei*	152,245	82,772	17,993	25,740	36.7	34.39	30.29	42.66	133	88	37	8
*Pilea basicordata*	152,220	82,706	17,994	25,760	36.69	34.38	30.24	42.64	132	87	37	8
*Pilea notata*	150,956	81,438	18,032	25,743	36.78	34.5	30.25	42.69	132	87	37	8
*Pilea angulata*	151,581	82,540	18,033	25,504	36.63	34.28	30.11	42.73	132	87	37	8
*Pilea sinofasciata*	152,283	83,250	18,385	25,324	36.63	34.23	30.5	42.8	131	86	37	8
*Pilea aquarum*	152,009	82,928	18,451	25,315	36.65	34.2	30.36	42.94	131	86	37	8
*Pilea peperomioides*	152,327	83,292	18,363	25,336	36.35	33.87	30.01	42.73	131	86	37	8
*Pilea plataniflora*	152,143	83,132	18,371	25,320	36.35	33.87	29.89	42.77	130	85	37	8
*Pilea sinocrassifolia*	151,462	82,722	18,406	25,167	36.7	34.33	30.61	42.82	131	86	37	8
*Pilea danxiaensis*	151,857	82,836	18,407	25,307	36.68	34.27	30.67	42.81	131	86	37	8
*Pilea pumila*	152,036	83,061	18,379	25,298	36.24	33.69	29.79	42.77	132	87	37	8
*Pilea microphylla*	150,019	82,478	17,275	25,133	36.61	34.14	30.78	42.66	132	87	37	8
*Pilea micromeriifolia*	143,738	89,128	17,888	18,361	36.54	34.36	30.6	44.74	127	82	37	8

**FIGURE 1:**
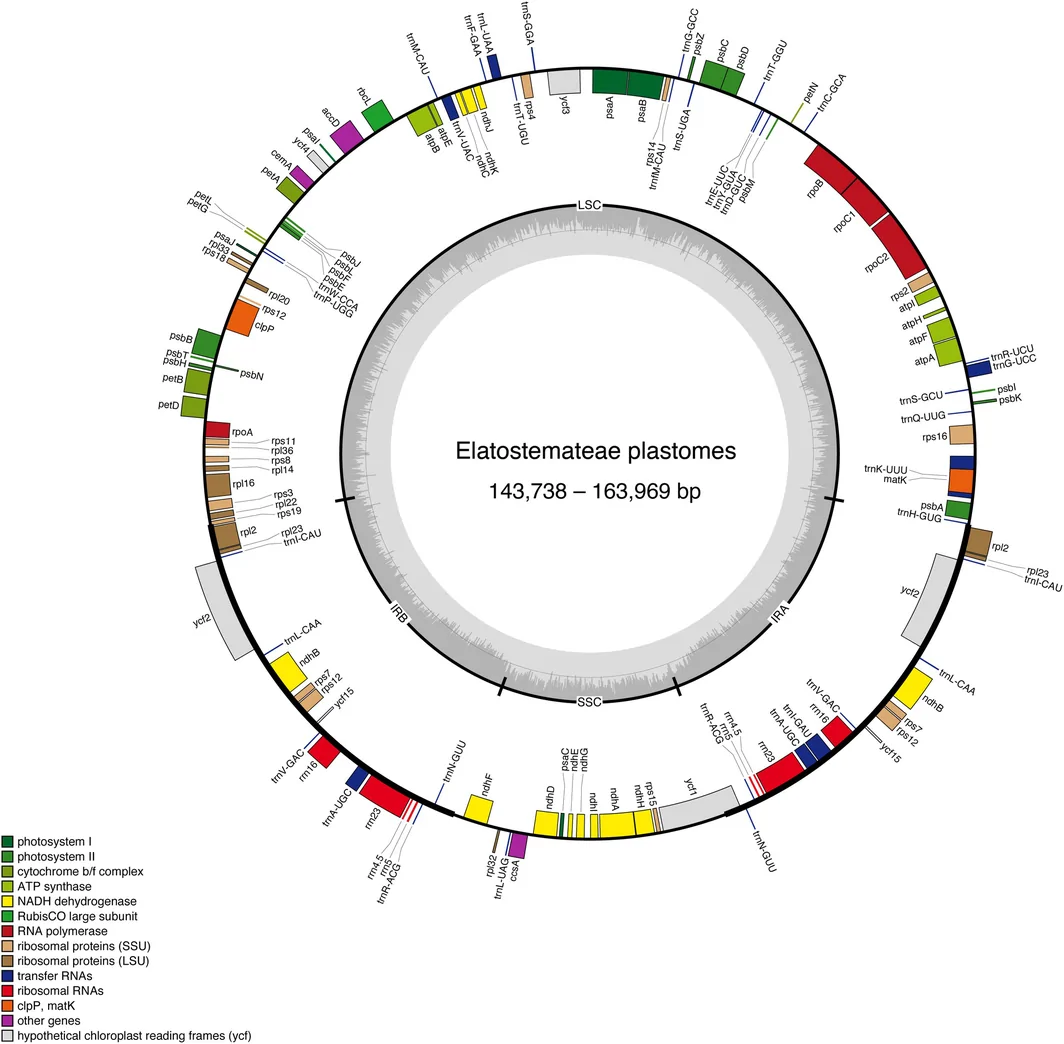
Gene maps of plastid genomes for Elatostemateae. The outer circles highlight the inverted repeats (IRs) with bold lines. The inner circles represent GC content across the genome, with lighter gray denoting AT content. The tribe's name and genome size are displayed at the center of each plot. Genes are color‐coded by functional group.

The overall genomic GC content presented structural stability across the investigated taxa, exhibiting a narrow fluctuation margin between 36.23% and 36.87%. Furthermore, specific region‐based GC analysis confirmed typical compositional biases. The dense IR regions maintained higher GC concentrations (41.04%–44.74%) compared to the relatively AT‐rich LSC (33.63%–34.60%) and SSC (29.59%–31.71%) zones. Computational annotation revealed a conserved genetic inventory, detecting between 127 and 133 distinct genes across the dataset. This suite included 82–88 specific protein‐coding genes (PCGs), a preservation of 37 functional tRNA genes, and 8 standardized rRNA genes. Structural analysis of individual genes across all 40 newly sequenced plastomes confirmed that exactly 15 genes possess a single functional intron (specifically *atpF*, *ndhA*, *ndhB*, *petB*, *petD*, *rpl2*, *rpl16*, *rpoC1*, *rps16*, *trnA‐UGC*, *trnG‐UCC*, *trnI‐GAU*, *trnK‐UUU*, *trnL‐UAA*, and *trnV‐UAC*), whereas two conserved genes (*clpP* and *ycf3*) functionally rely upon two distinct introns for proper expression.

### 3.2 Phylogenetic Patterns and Cytonuclear Concordance

Analyses of the CDS, CP, nrDNA, and concatenated CP + nrDNA datasets recovered the same three major Elatostemateae clades and the same principal backbone, although support differed among datasets (Figures 2 and 3). For example, Clade A received bootstrap/posterior‐probability support of 98.3/1.0 in the CP analysis and 98.1/1.0 in the nrDNA analysis, whereas Clade C was fully supported in the CP analysis and received 99.3/1.0 in the nrDNA analysis. Representative internal nrDNA nodes showed lower support, including 78.7/0.97 within *Elatostema* and 84.9/1.0 within *Pilea* (Figure 2B). Urticeae and Elatostemateae were recovered as sister lineages in the sampled family‐level framework.

**FIGURE 2:**
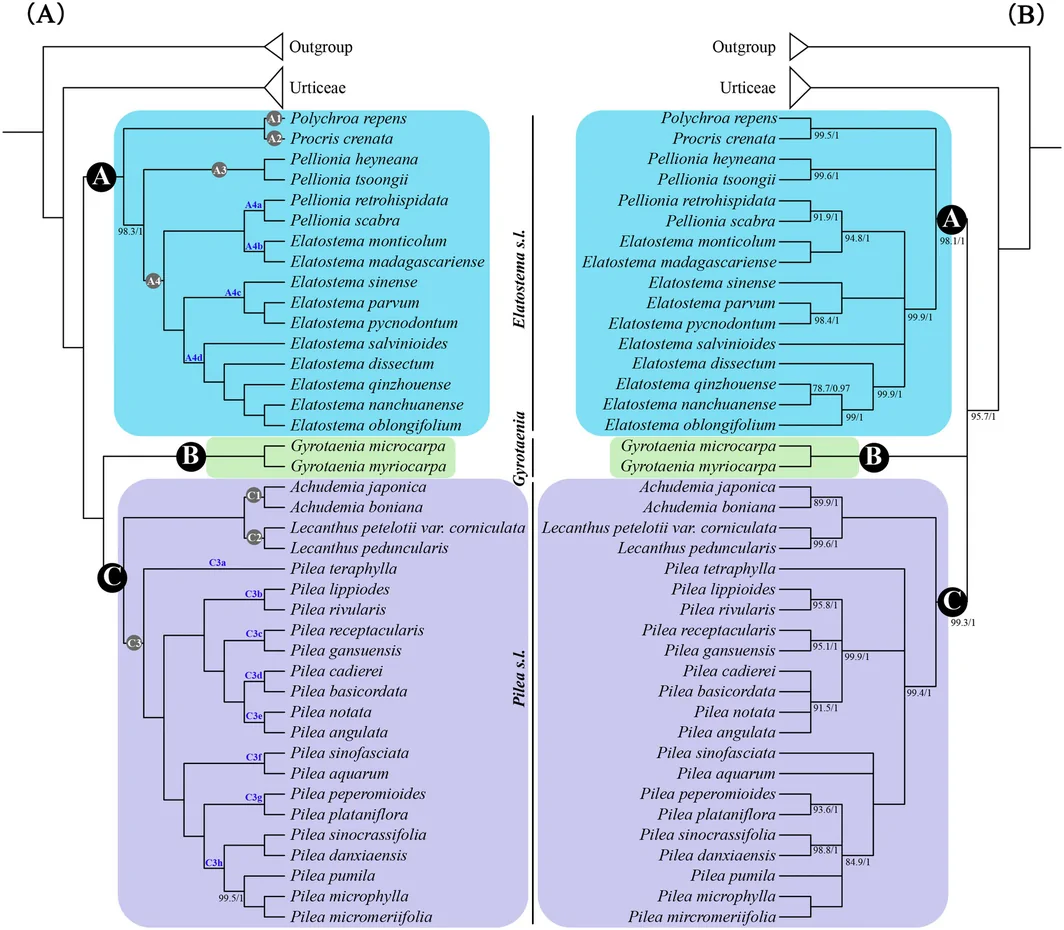
Comparisons of phylogenetic topologies of Elatostemateae, inferred by ML analyses of (A) CP genome, (B) nrDNA. Support values (bootstrap/posterior probability) are shown below branches; unlabeled nodes have full support (100/1.0).

**FIGURE 3:**
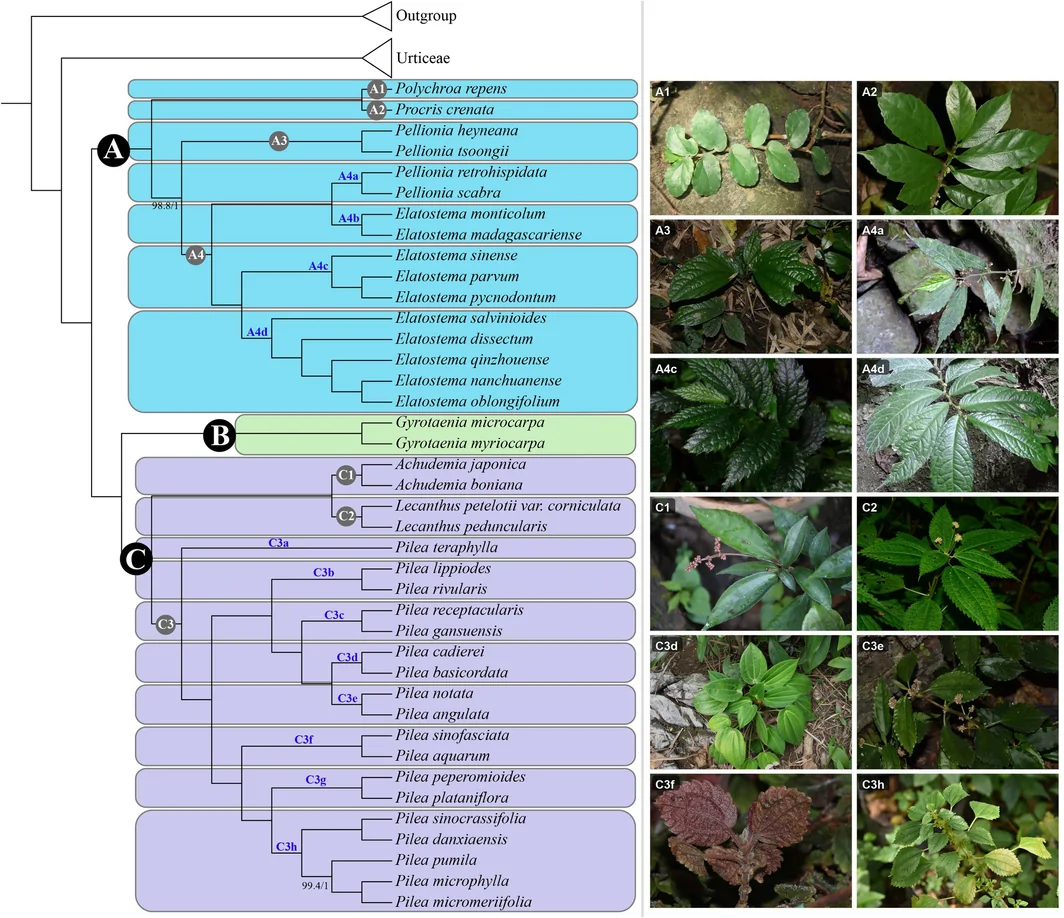
Phylogenomic tree of Elatostemateae, inferred from the combined CP genome + nrDNA dataset reconstructed by ML analysis. Support values (bootstrap/posterior probability) are shown below branches; unlabeled nodes have full support (100/1.0). Major clades are annotated according to Tseng et al. (2019) and Fu et al. (2022). The panels on the right display representative morphological photographs of species corresponding to the annotated subclades in the phylogenetic tree.

Within Elatostemateae, three principal clades were recovered: *Elatostema s.l*. (Clade A), *Gyrotaenia* (Clade B), and *Pilea s.l*. (Clade C). Within Clade A, four subclades (A1–A4) corresponded to the taxonomic groups *Polychroa*, *Procris*, *Elatostematoides*, and *Elatostema*, respectively. *Elatostema* (A4) was further divided into four subclades (A4a–A4d).

Clade C was recovered as sister to *Gyrotaenia* (Clade B). Within Clade C, three subclades (C1–C3) corresponded to *Achudemia*, *Lecanthus*, and *Pilea*, and *Pilea* (C3) contained eight subclades (C3a–C3h). The nrDNA topology therefore agreed with the plastid topology at the level of these major clades, while several internal nrDNA branches had reduced bootstrap support as noted above (Figure 2B). Representative habit photographs are mapped to selected molecular subclades in Figure 3 (A1, A2, A3, A4a, A4c, A4d, C1, C2, C3d, C3e, C3f, and C3h) to provide morphological context; these images were not used as characters in the phylogenetic reconstruction.

### 3.3 Plastome Rearrangements, IR Dynamics, and MT Integration

Whole‐plastome alignment showed that most sampled Elatostemateae plastomes were collinear, with several lineage‐specific structural variants. Specifically, *Elatostema parvum* (Blume) Blume ex Miq. and *Elatostema pycnodontum* W.T.Wang exhibited an approximately 20‐kb inversion within the LSC region (Figure 4), extending from *rpoB* to *trnQ‐UUG* and encompassing approximately 14 genes. These inversion‐bearing taxa form subclade A4c, whereas the PTMT‐bearing taxa described below occur in subclade A4d, allowing the major structural variants to be interpreted in their phylogenetic context (Figures 3 and 4).

**FIGURE 4:**
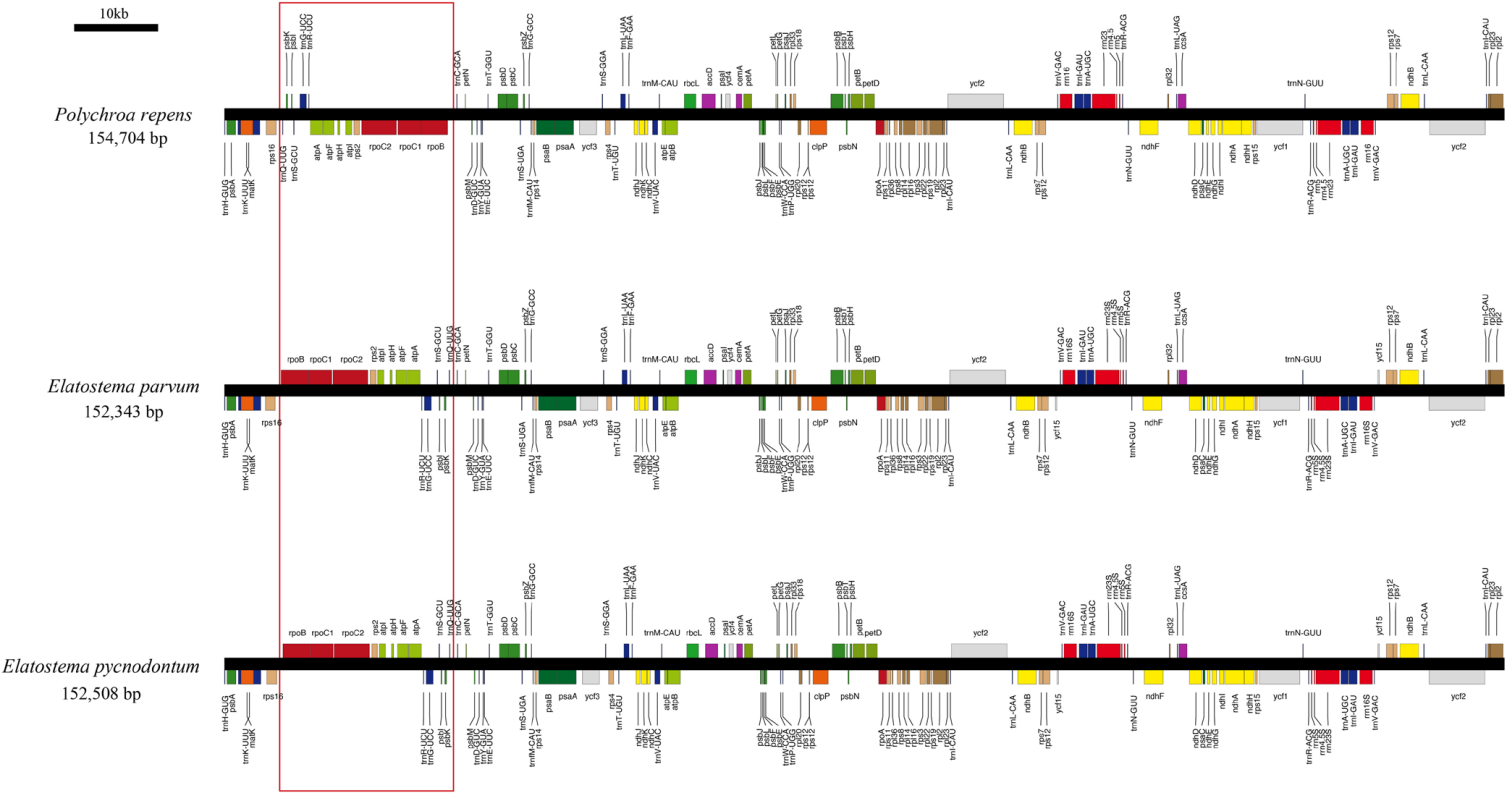
Comparative plastome maps illustrate an inversion in the LSC region (red box) of *Elatostema parvum* and *E. pycnodontum* relative to *Polychroa repens*, which represents the typical plastome structure. Gene colors denote functional groups, and arrows indicate transcription direction.

Assessment of IR junctions revealed substantial variation in the extent of IR expansion and contraction among Elatostemateae plastomes. A 3941‐7033‐bp insertion between *ycf15* and *trnV‐GAC* within the IR was detected in *Elatostema nanchuanense* W.T.Wang and *Elatostema oblongifolium*, producing markedly longer IR regions than in the other sampled taxa. The inserted sequence was subsequently evaluated as a putative plastid mitochondrial DNA‐derived sequence (PTMT).

BLASTn searches of the inserted *ycf15‐trnV‐GAC* sequence against the NCBI nucleotide database recovered matches to mitochondrial and plastid sequences. To verify that the insertion was not an assembly artifact, plastomes of the two affected species were assembled independently with GetOrganelle and Geneious, and the insertion boundaries were checked against read coverage (Figure 5). Together, the sequence similarity and coverage evidence support the presence of a genuine PTMT insertion in these plastomes.

**FIGURE 5:**
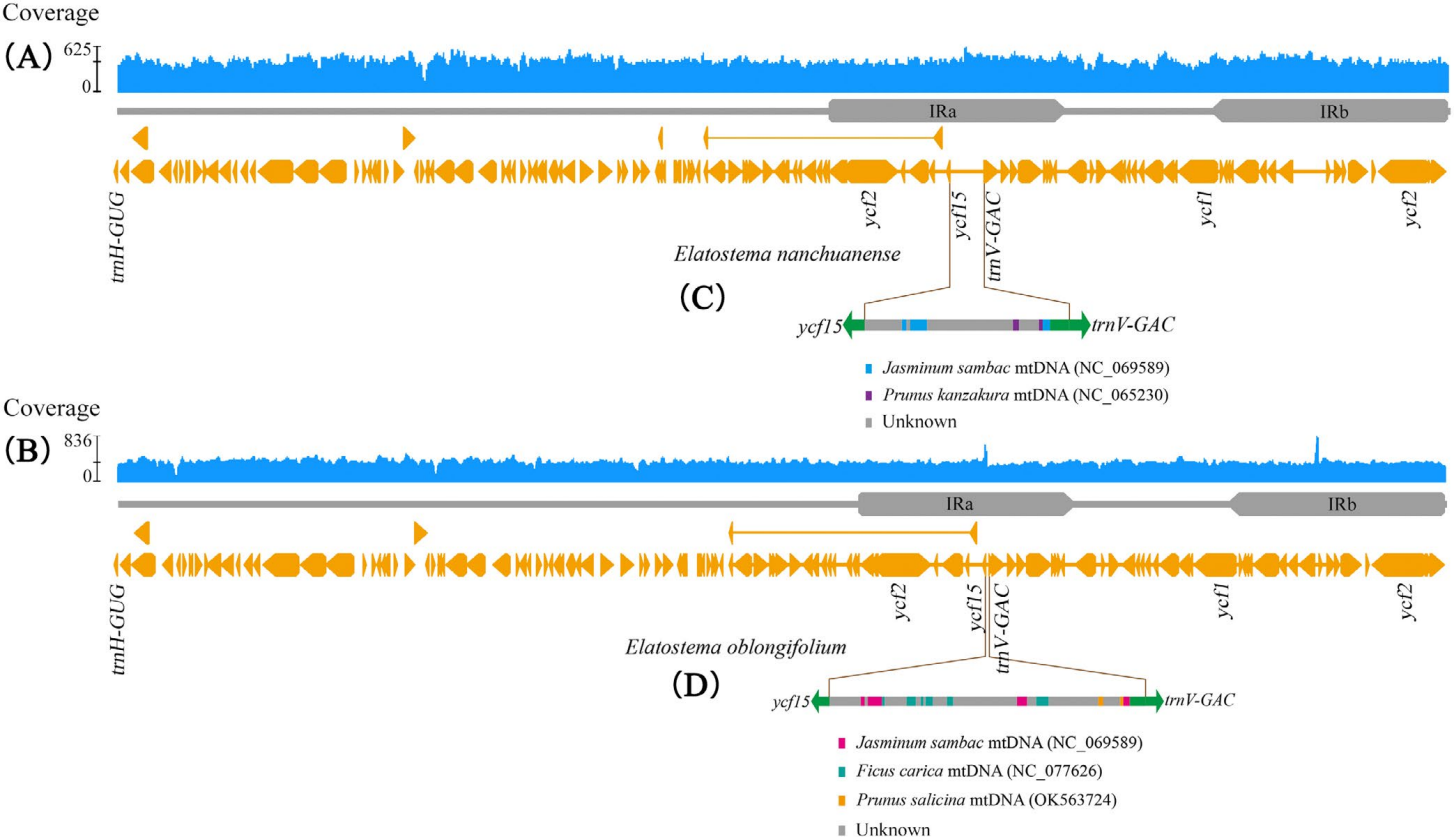
Schematic diagrams of PTMT integration in *Elatostema*. Analyses of the DNA‐seq read coverage across the whole plastome: (A) *E. nanchuanense*, (B) *E. oblongifolium*. Blast results of the integrated PTMT showing a mixture of fragments best matching mtDNAs of some taxa with color codes given below: (C) *E. nanchuanense*, (D) *E. oblongifolium*.

*Pilea tetraphylla* (Steud.) Blume showed the most pronounced asymmetric IR boundary shift (Figure S1). At JLB, IRb contraction shifted the boundary toward the IR and increased the LSC length, whereas expansion at JSB reduced the SSC length. These shifts moved *ycf2*, *trnI‐CAU*, *rpl23*, and rpl2 from the IR into the LSC, reducing them to single‐copy status, while *ycf1* and *rps15* were incorporated into the IR and became duplicated. Across the more conserved plastomes, JLB/JLA were associated primarily with *rps19‐rpl2* and rpl2‐trnH‐GUG, whereas JSB/JSA were associated with *ndhF* and *ycf1*/*trnN‐GUU*, respectively (Figure S1).

*Pilea micromeriifolia* showed a different pattern characterized by pronounced IR contraction. The shift moved *ycf2*, *trnI‐CAU*, *rpl23*, *rpl2*, and *rps19* from the IR into the LSC and reduced the IR by approximately 7 kb (Figure S1). Because the most unusual IR configurations are restricted to particular *Pilea* subclades, these junction patterns may provide useful phylogenetic characters, although broader sampling is required before they are used for taxonomic diagnosis.

### 3.4 Repetitive Sequences and Nucleotide Diversity

Across the 40 plastomes, 5477 repeat elements were identified: 3042 SSRs, 1379 dispersed long repeats, and 1056 tandem long repeats (Figure 6). This classification follows the methods above, separating short microsatellite repeats from longer dispersed and tandem repeats.

**FIGURE 6:**
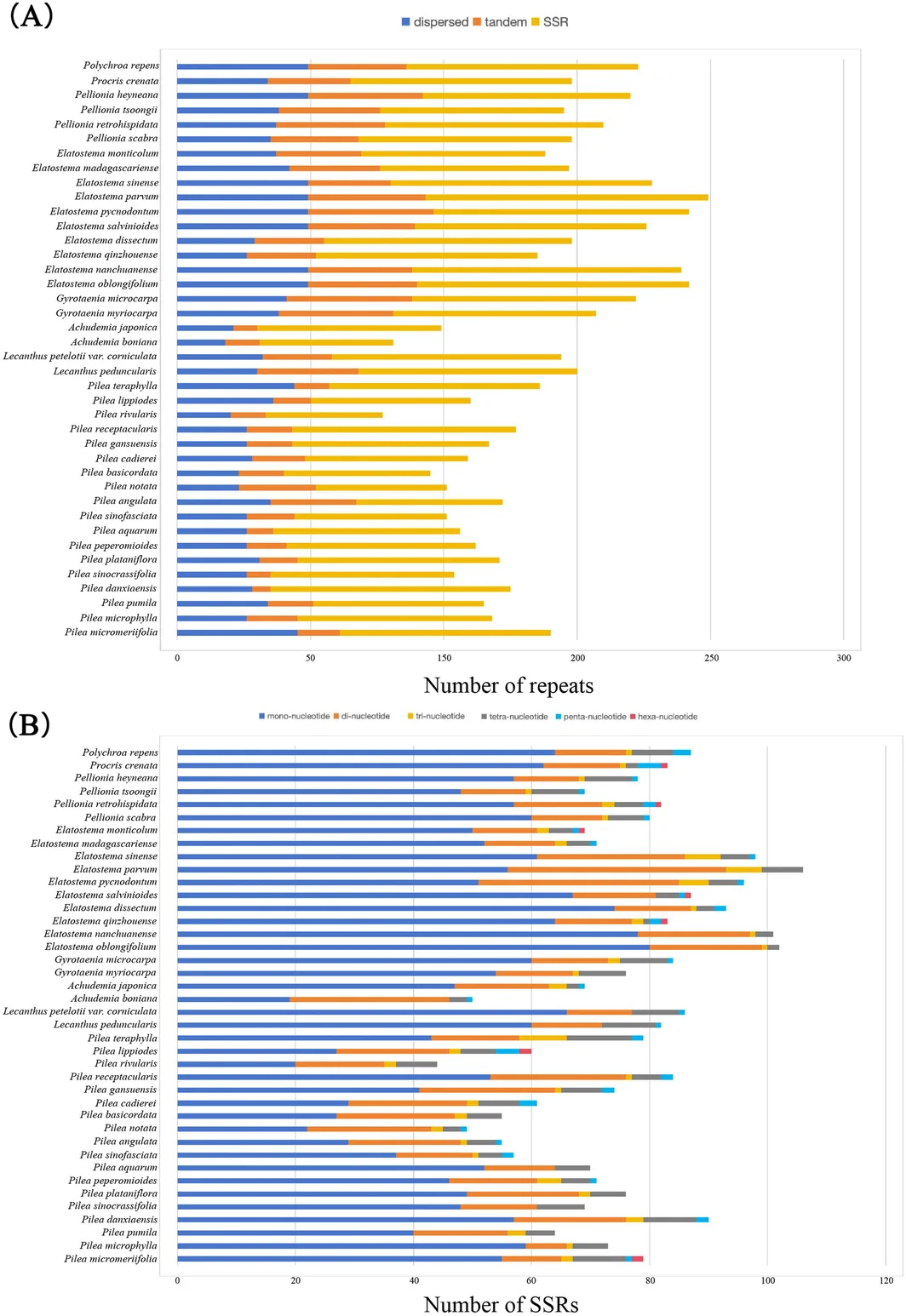
The maps of simple sequence repeats in Elatsotemateae. (A) The numbers of three types of repeats, (B) the number of SSRs of each of six repeat types, categorized by number of nucleotides.

The abundance of dispersed and tandem long repeats varied among species, from 7 in *Pilea danxiaensis* L.F.Fu, A.K.Monro & Y.G.Wei to 49 in *Polychroa repens*, *Elatostema* sinense H.Schroet., *Elatostema parvum*, *Elatostema pycnodontum*, *Elatostema salvinioides* W.T.Wang, *Elatostema nanchuanense*, and *Elatostema oblongifolium*. SSR abundance ranged from 44 in *Pilea rivularis* Wedd. to 106 in *Elatostema parvum*. Mononucleotide SSRs were most frequent (2021; 66.41%), dominated by poly‐A and poly‐T motifs (Figure 6B); di‐, tri‐, tetra‐, penta‐, and hexanucleotide SSRs accounted for 21.75%, 2.50%, 7.46%, 1.58%, and 0.30%, respectively.

Nucleotide‐diversity analyses showed that intergenic spacers were generally more variable than coding genes (Figure 7). Among the 100 genic regions, *ycf1*, *rpl22*, and *rps16* had Pi values > 0.08. Among the 95 intergenic spacers, *rps15‐ycf1*, *trnH‐GUG‐psbA*, *rpl32‐trnL‐UAG*, and *ndhF‐rpl32* had Pi values > 0.16. These regions represent localized peaks of sequence variation and are therefore candidates for marker development; they should not be interpreted as causal drivers of plastome evolution.

**FIGURE 7:**
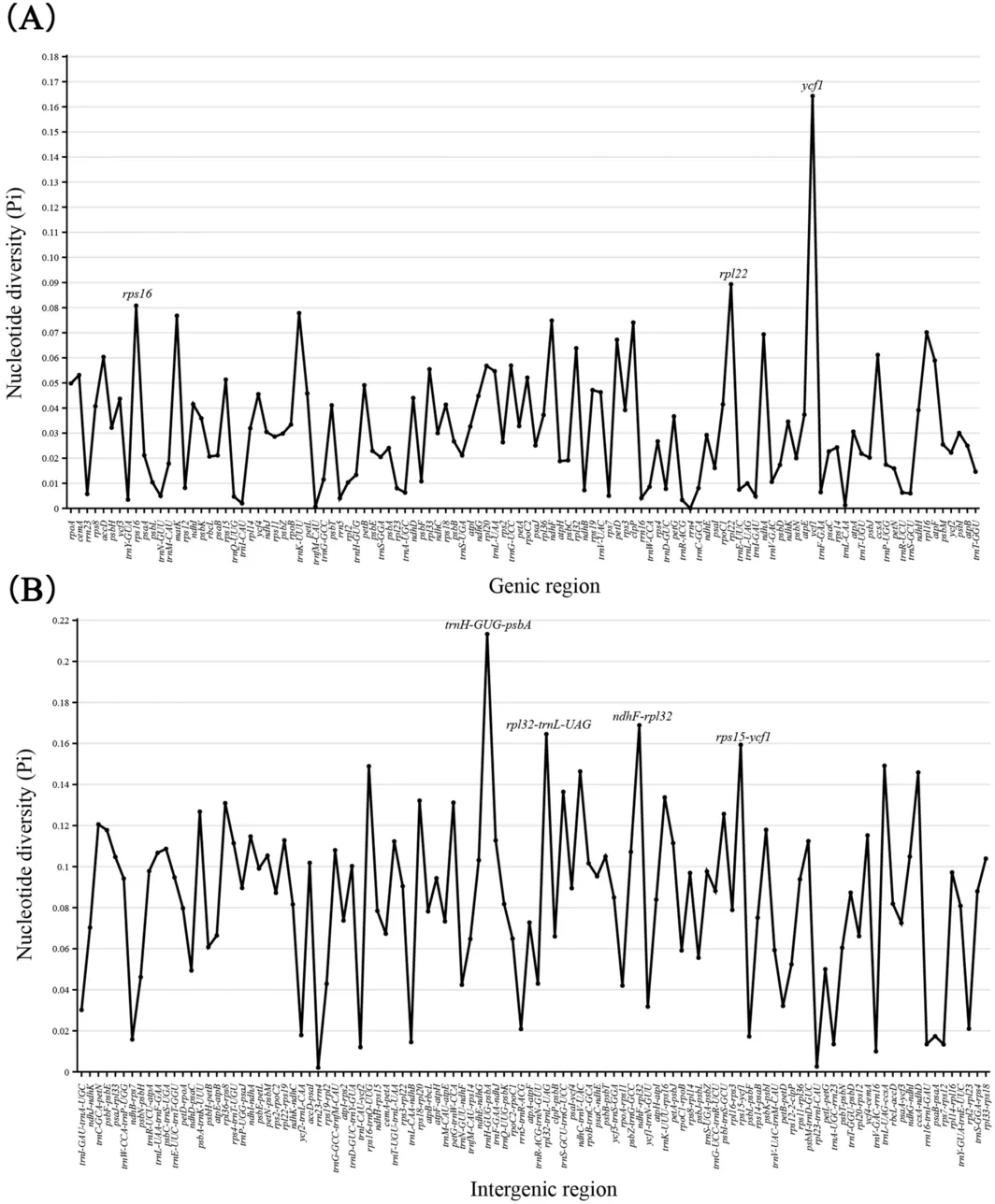
The nucleotide variability (Pi) value in Elatostemateae. (A) The Pi value of genic regions (B) The Pi value of intergenic regions.

## 4 Discussion

### 4.1 Cytonuclear Discordance and Generic Phylogeny

This study provides a multi‐locus phylogenomic assessment of Elatostemateae supported by broad taxon sampling and whole‐plastome plus nrDNA datasets. The plastid and nrDNA analyses recovered the same major clades, while the concatenated CP + nrDNA dataset provided a well‐resolved summary topology. The reduced support at several internal nrDNA nodes (Figure 2B) nevertheless shows that congruence is strongest at the major‐clade level rather than at every internal branch.

The placement of *Gyrotaenia* is the clearest point of cytonuclear discordance. Our plastid and nrDNA analyses recover *Gyrotaenia* within Elatostemateae as sister to *Pilea s.l*. (Figure 8A,B), consistent with earlier multi‐locus results (Wu et al. 2013). In contrast, Angiosperms353 low‐copy nuclear genes place *Gyrotaenia* together with *Myriocarpa* as sister to core Elatostemateae (*Elatostema s.l*. + *Pilea s.l*.) (Figure 8D; Monro et al. 2025). Monro et al. (2025) reported high local posterior probability (LPP = 0.91) but low normalized quartet support (NQS = 39.95%) for the core Elatostemateae clade, indicating substantial gene‐tree conflict despite the high LPP. A separate nine‐locus Sanger analysis constrained by the Angiosperms353 topology also placed *Gyrotaenia* + *Myriocarpa* outside core Elatostemateae (Figure 8C).

**FIGURE 8:**
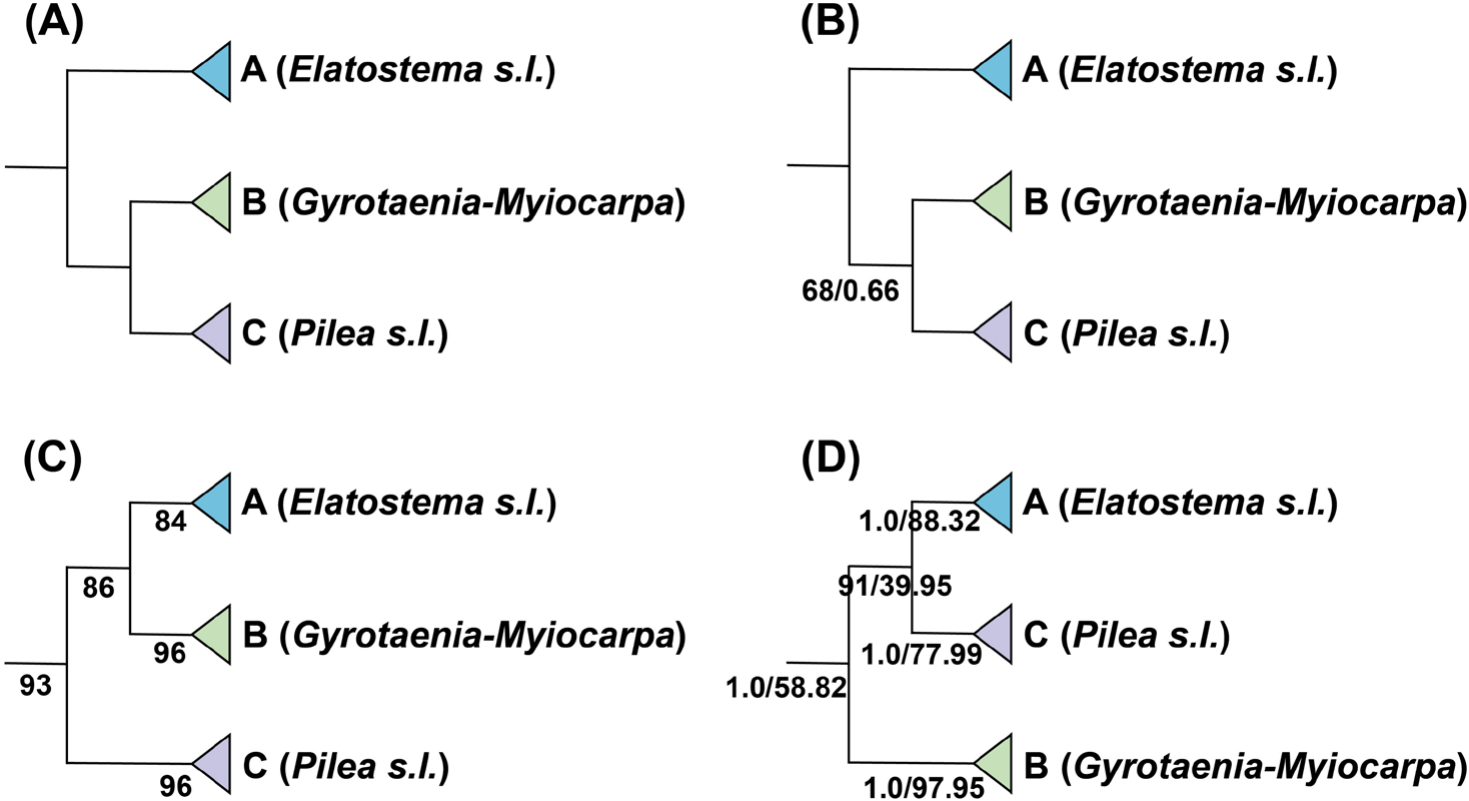
Phylogenetic relationships of *Elatostema s.l*., *Pilea s.l*., and *Gyrotaenia*‐*Myriocarpa* based on different molecular datasets. (A) CP genome + nrDNA dataset from this study. (B) Seven‐locus Sanger dataset from Wu et al. (2013). (C) Nine‐locus Sanger dataset from Monro et al. (2025), constrained by the Angiosperms353 HybSeq nuclear‐gene topology. (D) Angiosperms353 HybSeq low‐copy nuclear‐gene dataset from Monro et al. (2025). Support values are shown below branches. For (A–C), values indicate bootstrap percentages/posterior probabilities, with unlabeled nodes representing full support (100/1.0). For (D), values indicate local posterior probability (LPP)/normalized quartet support (NQS, %). Thus, values such as 1.0/58.82 report two different support metrics rather than a duplicated bootstrap/posterior‐probability pair.

The discordance between plastid and low‐copy nuclear topologies can be explained by more than one evolutionary process. Incomplete lineage sorting (ILS) during rapid divergence and historical introgression or chloroplast capture are both plausible, non‐exclusive hypotheses, but the present plastome + nrDNA data cannot distinguish between them. Accordingly, we interpret ILS and introgression as alternative explanations rather than demonstrated causes. Denser sampling of independent low‐copy nuclear loci or whole‐genome data will be required to test these hypotheses and quantify historical gene flow. These alternative processes are well documented sources of gene‐tree and cytonuclear discordance in plants (Soltis and Kuzoff 1995; Maddison and Knowles 2006; Lee‐Yaw et al. 2019; Guo et al. 2023).

In parallel with resolving deeper relationships, the phylogenomic results are broadly consistent with previous morphology‐based taxonomic revisions. *Elatostema*, *Elatostematoides*, and *Procris* are recovered within *Elatostema s.l*. as in Tseng et al. (2019), whereas the sampled species historically assigned to *Pellionia* occur in multiple lineages (Figure 2), supporting continued reassessment of that generic boundary. *Polychroa repens*, historically treated as *Pellionia repens*, is allied to *Procris*, consistent with earlier analyses (Tseng et al. 2019; Monro et al. 2025; Tseng et al. 2025). The recovered relationship among *Pilea*, *Achudemia*, and *Lecanthus* is also compatible with the morphology‐based recognition of *Achudemia*, including diagnostic floral and achene characters (Fu et al. 2022).

### 4.2 Plastome Structural Evolution

Elatostemateae plastomes show pronounced structural variation, especially in IR boundaries. Variation in plastome size across seed plants is commonly associated with expansion and contraction of IR regions (Jansen and Ruhlman 2012), and our data show the same general pattern. *Pilea tetraphylla* exhibits an unusually asymmetric combination of IR contraction and expansion, with opposite effects on LSC and SSC lengths. Similar asymmetric IR shifts have been reported in other angiosperm lineages and can arise through double‐strand‐break repair and illegitimate recombination near repeat boundaries (Abdullah et al. 2020; Wang et al. 2017).

Large inversions of approximately 20 kb were also detected in the plastomes of *Elatostema pycnodontum* and *Elatostema parvum*. Large plastome inversions occur in several angiosperm families (Charboneau et al. 2021; Lee et al. 2021; Röschenbleck et al. 2017), but these are the first documented examples in our Elatostemateae sampling. The inversion breakpoints lie near *trnC‐GCA* and *trnQ‐UUG*. The proximity of tRNA loci is consistent with recombination‐prone regions reported in other plastomes, but it does not by itself demonstrate that tRNA genes mechanically caused the inversion (Hiratsuka et al. 1989; Walker et al. 2014).

### 4.3 Putative Origin of the PTMT Insertion

A notable structural feature is the plastid mitochondrial DNA‐derived sequence (PTMT) detected in two *Elatostema* plastomes (Figure 5). Transfer of organellar DNA to the nucleus is common, whereas mitochondrial‐to‐plastid transfer is comparatively rare, with documented examples in several distantly related angiosperm lineages (Raman et al. 2021; Wu, Chen, and Chaw 2022).

The PTMT insertion likely reflects a rare historical recombination event. One plausible scenario is that mitochondrial DNA was incorporated during repair of a plastome double‐strand break, followed by subsequent sequence loss and stabilization of the retained fragment. The present sequence between *ycf15* and *trnV‐GAC* may therefore represent a remnant of a larger ancestral insertion (Figure 5; Wu, Chen, and Chaw 2022). Because the event is restricted to a subset of *Elatostema* in our sampling, it may be phylogenetically informative, but its exact age and mechanism cannot be inferred from the current plastome data alone.

### 4.4 Biogeographic Context of Plastome Structural Variation

A key observation from this genomic survey is that the major plastome structural variants are phylogenetically clustered rather than randomly distributed across the sampled tree. The LSC inversion, PTMT insertion, pronounced IR contraction, and asymmetric IR shift are associated with subclades A4c, A4d, C3h, and C3a, respectively (Figures 3, 4, 5, and S1). These clades also occupy different geographic regions, including Indomalayan, Neotropical, and African areas, providing a biogeographic context for the structural variation.

The present sampling does not demonstrate that geographic isolation or ecological conditions caused these plastome rearrangements. Historical isolation, demographic change, recombination, and stochastic fixation could all contribute to the observed distribution of structural variants. We therefore treat the apparent association between phylogeny, geography, and plastome structure as a hypothesis for future testing rather than evidence of a specific bottleneck‐driven mechanism. Population‐level sampling and independent nuclear data will be required to evaluate whether any structural variant is associated with demography, geography, or selection.

## 5 Conclusion

This study establishes a multi‐locus phylogenomic framework for Elatostemateae by integrating complete plastomes and nrDNA across broad taxonomic sampling. The analyses resolve the major sampled clades and support several recent taxonomic realignments, while the placement of *Gyrotaenia* highlights a cytonuclear conflict that may reflect incomplete lineage sorting or historical introgression. Comparative plastome analyses further reveal large inversions, substantial IR‐boundary shifts, and a rare PTMT insertion, demonstrating pronounced structural variation within the tribe. Seven highly variable regions, including ycf1, are promising candidates for DNA barcoding and species‐level studies. Together, these results clarify the evolutionary history of the sampled lineages while identifying nuclear genomic sampling and broader population‐level comparisons as priorities for future work.

## Author Contributions

**Long‐Fei Fu:** conceptualization (equal), data curation (equal), investigation (equal), writing – original draft (equal). **Zi‐Bing Xin:** data curation (equal), writing – original draft (equal). **Chi Xiong:** data curation (equal), investigation (equal). **Fang Wen:** data curation (equal). **Yi‐Gang Wei:** conceptualization (equal), funding acquisition (equal), supervision (equal). **Alexandre K. Monro:** conceptualization (equal), supervision (equal), writing – review and editing (equal).

## Funding

This work was supported by specific targeted funding originating from the Fund of Guangxi Key Laboratory of Plant Conservation and Restoration Ecology in Karst Terrain (Grant No. 22‐035‐26), the Basic Research Fund of Guangxi Academy of Sciences (Grant No. CQZ‐C‐1901), the National Natural Science Foundation of China (Grant Nos. 32360297 and 32460302) and the Light of West China Program administered by the Chinese Academy of Sciences (Grant No. 82).

## Conflicts of Interest

The authors declare no conflicts of interest.

## Supporting information


**Figure S1:** Comparison of the borders of large single‐copy (LSC), small single‐copy (SSC), and inverted repeat (IR) regions among Elatostemateae species.

**Table S1:** Substitution models used for different datasets and tree reconstruction methods.

## Data Availability

The sequence data supporting the findings of this study are openly available in the National Center for Biotechnology Information (NCBI; https://www.ncbi.nlm.nih.gov/) GenBank database. Accession numbers for all plastome and nrDNA sequences, including all newly generated sequences, are provided in Table 1.

## References

[ece374416-bib-0001] Abdullah , C. L. Henriquez , F. Mehmood , et al. 2020. “Complete Chloroplast Genomes of Anthurium Huixtlense and Pothos Scandens (Pothoideae, Araceae): Unique Inverted Repeat Expansion and Contraction Affect Rate of Evolution.” Journal of Molecular Evolution 88: 562–574. 10.1007/s00239-020-09958-w PMC7445159 32642873

[ece374416-bib-0002] Aho, K. , D. Derryberry , and T. Peterson . 2014. “Model Selection for Ecologists: The Worldviews of AIC and BIC.” Ecology 95: 631–636. 10.1890/13-1452.1 24804445

[ece374416-bib-0003] Benson, G. 1999. “Tandem Repeats Finder: A Program to Analyze DNA Sequences.” Nucleic Acids Research 27: 573–580. 10.1093/nar/27.2.573 PMC148217 9862982

[ece374416-bib-0004] Capella‐Gutiérrez, S. , J. M. Silla‐Martínez , and T. Gabaldón . 2009. “trimAl: A Tool for Automated Alignment Trimming in Large‐Scale Phylogenetic Analyses.” Bioinformatics 25: 1972–1973. 10.1093/bioinformatics/btp348 PMC2712344 19505945

[ece374416-bib-0005] Charboneau, J. L. , R. C. Cronn , A. Liston , M. F. Wojciechowski , and M. J. Sanderson . 2021. “Plastome Structural Evolution and Homoplastic Inversions in Neo‐Astragalus (Fabaceae).” Genome Biology and Evolution 13: evab215. 10.1093/gbe/evab215 PMC8486006 34534296

[ece374416-bib-0008] Chen, L. Y. , M. S. Song , H. G. Zha , and Z. M. Li . 2014. “A Modified Protocol for Plant Genome DNA Extraction.” Plant Diversity and Resources 36: 375–380.

[ece374416-bib-0009] Choi, I. S. , and B. H. Choi . 2017. “The Distinct Plastid Genome Structure of *Maackia fauriei* (Fabaceae: Papilionoideae) and Its Systematic Implications for Genistoids and Tribe Sophoreae.” PLoS One 12: e0173766. 10.1371/journal.pone.0173766 PMC5388331 28399123

[ece374416-bib-0010] Darling, A. C. , B. Mau , F. R. Blattner , and N. T. Perna . 2004. “Mauve: Multiple Alignment of Conserved Genomic Sequence With Rearrangements.” Genome Research 14: 1394–1403. 10.1101/gr.2289704 PMC442156 15231754

[ece374416-bib-0011] Darriba, D. , G. L. Taboada , R. Doallo , and D. Posada . 2012. “jModelTest 2: More Models, New Heuristics and Parallel Computing.” Nature Methods 9: 772. 10.1038/nmeth.2109 PMC4594756 22847109

[ece374416-bib-0012] Dugas, D. V. , D. Hernandez , E. J. Koenen , et al. 2015. “Mimosoid Legume Plastome Evolution: IR Expansion, Tandem Repeat Expansions and Accelerated Rate of Evolution in *clpP* .” Scientific Reports 5: 16958. 10.1038/srep16958 PMC4655330 26592928

[ece374416-bib-0013] Friis, I. 1989. “The Urticaceae: A Systematic Review.” In Evolution, Systematics, and Fossil History of the Hamamelidae, edited by P. R. Crane and S. Blackmore , vol. 2, 285–308. Clarendon Press.

[ece374416-bib-0014] Fu, L. , A. K. Monro , T. Yang , et al. 2021. “ *Elatostema qinzhouense* (Urticaceae), a New Species From Limestone Karst in Guangxi, China.” PeerJ 9: e11148. 10.7717/peerj.11148 PMC8061576 33976965

[ece374416-bib-0015] Fu, L. F. , F. Wen , O. Maurin , et al. 2022. “A Revised Delimitation of the Species‐Rich Genus Pilea (Urticaceae) Supports the Resurrection of Achudemia and a New Infrageneric Classification.” Taxon 71: 796–813.

[ece374416-bib-0017] Greiner, S. , P. Lehwark , and R. Bock . 2019. “OrganellarGenomeDRAW (OGDRAW) Version 1.3.1: Expanded Toolkit for the Graphical Visualization of Organellar Genomes.” Nucleic Acids Research 47: W59–W64. 10.1093/nar/gkz238 PMC6602502 30949694

[ece374416-bib-0018] Guo, C. , Y. Luo , L. M. Gao , et al. 2023. “Phylogenomics and the Flowering Plant Tree of Life.” Journal of Integrative Plant Biology 65: 299–323. 10.1111/jipb.13415 36416284

[ece374416-bib-0019] Hiratsuka, J. , H. Shimada , R. Whittier , et al. 1989. “The Complete Sequence of the Rice ( *Oryza sativa* ) Chloroplast Genome: Intermolecular Recombination Between Distinct tRNA Genes Accounts for a Major Plastid DNA Inversion During the Evolution of the Cereals.” Molecular and General Genetics 217: 185–194. 10.1007/BF02464880 2770692

[ece374416-bib-0022] Jansen, R. K. , and T. A. Ruhlman . 2012. “Plastid Genomes of Seed Plants.” Genomics of Chloroplasts and Mitochondria 35: 103–126.

[ece374416-bib-0023] Jin, J. J. , W. B. Yu , J. B. Yang , et al. 2020. “GetOrganelle: A Fast and Versatile Toolkit for Accurate De Novo Assembly of Organelle Genomes.” Genome Biology 21: 1–31. 10.1186/s13059-020-02154-5 PMC7488116 32912315

[ece374416-bib-0024] Katoh, K. , and D. M. Standley . 2013. “MAFFT Multiple Sequence Alignment Software Version 7: Improvements in Performance and Usability.” Molecular Biology and Evolution 30: 772–780. 10.1093/molbev/mst010 PMC3603318 23329690

[ece374416-bib-0025] Kearse, M. , R. Moir , A. Wilson , et al. 2012. “Geneious Basic: An Integrated and Extendable Desktop Software Platform for the Organization and Analysis of Sequence Data.” Bioinformatics 28: 1647–1649. 10.1093/bioinformatics/bts199 PMC3371832 22543367

[ece374416-bib-0026] Kim, C. , T. Deng , M. Chase , D. G. Zhang , Z. L. Nie , and H. Sun . 2015. “Generic Phylogeny and Character Evolution in Urticeae (Urticaceae) Inferred From Nuclear and Plastid DNA Regions.” Taxon 64: 65–78.

[ece374416-bib-0028] Kurtz, S. , J. V. Choudhuri , E. Ohlebusch , C. Schleiermacher , J. Stoye , and R. Giegerich . 2001. “REPuter: The Manifold Applications of Repeat Analysis on a Genomic Scale.” Nucleic Acids Research 29: 4633–4642. 10.1093/nar/29.22.4633 PMC92531 11713313

[ece374416-bib-0029] Lee, C. , I. S. Choi , D. Cardoso , et al. 2021. “The Chicken or the Egg? Plastome Evolution and an Independent Loss of the Inverted Repeat in Papilionoid Legumes.” Plant Journal 107: 861–875. 10.1111/tpj.15351 34021942

[ece374416-bib-0030] Lee‐Yaw, J. A. , C. J. Grassa , S. Joly , R. L. Andrew , and L. H. Rieseberg . 2019. “An Evaluation of Alternative Explanations for Widespread Cytonuclear Discordance in Annual Sunflowers (Helianthus).” New Phytologist 221: 515–526. 10.1111/nph.15386 30136727

[ece374416-bib-0031] Letunic, I. , and P. Bork . 2021. “Interactive Tree of Life (iTOL) v5: An Online Tool for Phylogenetic Tree Display and Annotation.” Nucleic Acids Research 49: W293–W296. 10.1093/nar/gkab301 PMC8265157 33885785

[ece374416-bib-0032] Li, H. , Q. Guo , and L. Xu . 2023. “CPJSdraw: Analysis and Visualization of Junction Sites of Chloroplast Genomes.” PeerJ 11: e15326. 10.7717/peerj.15326 PMC10182761 37193025

[ece374416-bib-0033] Li, J. L. , J. M. Tang , S. Y. Zeng , F. Han , J. Yuan , and J. Yu . 2021. “Comparative Plastid Genomics of Four Pilea (Urticaceae) Species: Insight Into Interspecific Plastid Genome Diversity in Pilea.” BMC Plant Biology 21: 25. 10.1186/s12870-020-02793-7 PMC7792329 33413130

[ece374416-bib-0036] Maddison, W. P. , and L. L. Knowles . 2006. “Inferring Phylogeny Despite Incomplete Lineage Sorting.” Systematic Biology 55: 21–30. 10.1080/10635150500354928 16507521

[ece374416-bib-0072] Miller, M. A. , W. Pfeiffer , and T. Schwartz . 2011. “The CIPRES Science Gateway: A Community Resource for Phylogenetic Analyses.” In Proceedings of the 2011 TeraGrid Conference: Extreme Digital Discovery, 1–8. Association for Computing Machinery.

[ece374416-bib-0039] Monro, A. K. 2006. “The Revision of Species‐Rich Genera: A Phylogenetic Framework for the Strategic Revision of Pilea (Urticaceae) Based on cpDNA, nrDNA, and Morphology.” American Journal of Botany 93: 426–441. 10.3732/ajb.93.3.426 21646202

[ece374416-bib-0040] Monro, A. K. , N. Bystriakova , L. Fu , F. Wen , and Y. Wei . 2018. “Discovery of a Diverse Cave Flora in China.” PLoS One 13: e0190801. 10.1371/journal.pone.0190801 PMC5802439 29415039

[ece374416-bib-0041] Monro, A. K. , O. Maurin , L. F. Fu , et al. 2025. “Classification of Urticaceae Based on Morphology and Phylogenetic Inference [Preprint].” *bioRxiv*. 10.1101/2025.05.16.651835.

[ece374416-bib-0042] Nguyen, L. T. , H. A. Schmidt , A. Von Haeseler , and B. Q. Minh . 2015. “IQ‐TREE: A Fast and Effective Stochastic Algorithm for Estimating Maximum‐Likelihood Phylogenies.” Molecular Biology and Evolution 32: 268–274. 10.1093/molbev/msu300 PMC4271533 25371430

[ece374416-bib-0043] Ogoma, C. A. , J. Liu , G. W. Stull , et al. 2022. “Deep Insights Into the Plastome Evolution and Phylogenetic Relationships of the Tribe Urticeae (Family Urticaceae).” Frontiers in Plant Science 13: 870949. 10.3389/fpls.2022.870949 PMC9164014 35668809

[ece374416-bib-0044] Qu, X. J. , M. J. Moore , D. Z. Li , and T. S. Yi . 2019. “PGA: A Software Package for Rapid, Accurate, and Flexible Batch Annotation of Plastomes.” Plant Methods 15: 1–12. 10.1186/s13007-019-0435-7 PMC6528300 31139240

[ece374416-bib-0047] Raman, G. , E. M. Lee , and S. Park . 2021. “Intracellular DNA Transfer Events Restricted to the Genus Convallaria Within the Asparagaceae Family: Possible Mechanisms and Potential as Genetic Markers for Biographical Studies.” Genomics 113: 2906–2918. 10.1016/j.ygeno.2021.06.033 34182083

[ece374416-bib-0048] Ronquist, F. , M. Teslenko , P. Van Der Mark , et al. 2012. “MrBayes 3.2: Efficient Bayesian Phylogenetic Inference and Model Choice Across a Large Model Space.” Systematic Biology 61: 539–542. 10.1093/sysbio/sys029 PMC3329765 22357727

[ece374416-bib-0049] Röschenbleck, J. , S. Wicke , S. Weinl , J. Kudla , and K. F. Müller . 2017. “Genus‐Wide Screening Reveals Four Distinct Types of Structural Plastid Genome Organization in Pelargonium (Geraniaceae).” Genome Biology and Evolution 9: 64–76. 10.1093/gbe/evw271 PMC5381562 28172771

[ece374416-bib-0050] Rozas, J. , A. Ferrer‐Mata , J. C. Sánchez‐DelBarrio , et al. 2017. “DnaSP 6: DNA Sequence Polymorphism Analysis of Large Data Sets.” Molecular Biology and Evolution 34: 3299–3302. 10.1093/molbev/msx248 29029172

[ece374416-bib-0052] Shi, L. , H. Chen , M. Jiang , et al. 2019. “CPGAVAS2, an Integrated Plastome Sequence Annotator and Analyzer.” Nucleic Acids Research 47: W65–W73. 10.1093/nar/gkz345 PMC6602467 31066451

[ece374416-bib-0053] Sinn, B. T. , D. D. Sedmak , L. M. Kelly , and J. V. Freudenstein . 2018. “Total Duplication of the Small Single Copy Region in the Angiosperm Plastome: Rearrangement and Inverted Repeat Instability in Asarum.” American Journal of Botany 105: 71–84. 10.1002/ajb2.1001 29532923

[ece374416-bib-0054] Soltis, D. E. , and R. K. Kuzoff . 1995. “Discordance Between Nuclear and Chloroplast Phylogenies in the Heuchera Group (Saxifragaceae).” Evolution 49: 727–742. 10.1111/j.1558-5646.1995.tb02309.x 28565145

[ece374416-bib-0056] Thiel, T. , W. Michalek , R. Varshney , and A. Graner . 2003. “Exploiting EST Databases for the Development and Characterization of Gene‐Derived SSR‐Markers in Barley ( *Hordeum vulgare* L.).” Theoretical and Applied Genetics 106: 411–422. 10.1007/s00122-002-1031-0 12589540

[ece374416-bib-0057] Tseng, Y. H. , H. C. Chien , and G. X. Zhu . 2025. “Comparative Plastome Analyses and Phylogenetic Insights of Elatostema.” BMC Plant Biology 25: 537. 10.1186/s12870-025-06569-9 PMC12032637 40281442

[ece374416-bib-0058] Tseng, Y. H. , A. K. Monro , Y. G. Wei , and J. M. Hu . 2019. “Molecular Phylogeny and Morphology of Elatostema s.l. (Urticaceae): Implications for Inter‐ and Infrageneric Classifications.” Molecular Phylogenetics and Evolution 132: 251–264. 10.1016/j.ympev.2018.11.016 30503949

[ece374416-bib-0059] Twyford, A. D. , and R. W. Ness . 2017. “Strategies for Complete Plastid Genome Sequencing.” Molecular Ecology Resources 17: 858–868. 10.1111/1755-0998.12626 PMC6849563 27790830

[ece374416-bib-0060] Villarreal, J. C. , L. L. Forrest , N. Wickett , and B. Goffinet . 2013. “The Plastid Genome of the Hornwort *Nothoceros aenigmaticus* (Dendrocerotaceae): Phylogenetic Signal in Inverted Repeat Expansion, Pseudogenization, and Intron Gain.” American Journal of Botany 100: 467–477. 10.3732/ajb.1200429 23416362

[ece374416-bib-0061] Walker, B. J. , T. Abeel , T. Shea , et al. 2014. “Pilon: An Integrated Tool for Comprehensive Microbial Variant Detection and Genome Assembly Improvement.” PLoS One 9: e112963. 10.1371/journal.pone.0112963 PMC4237348 25409509

[ece374416-bib-0062] Wang, R. N. , R. I. Milne , X. Y. Du , J. Liu , and Z. Y. Wu . 2020. “Characteristics and Mutational Hotspots of Plastomes in Debregeasia (Urticaceae).” Frontiers in Genetics 11: 729. 10.3389/fgene.2020.00729 PMC7360830 32733543

[ece374416-bib-0063] Wang, W. T. 2016. “Metapilea, a New Genus of Urticaceae From Guangxi.” Bulletin of Botanical Research 36: 164.

[ece374416-bib-0064] Wang, Y. , C. Xu , X. Guo , et al. 2024. “Phylogenomics Analysis of Scutellaria (Lamiaceae) of the World.” BMC Biology 22: 185. 10.1186/s12915-024-01982-2 PMC11367873 39218872

[ece374416-bib-0065] Wang, Y. H. , X. J. Qu , S. Y. Chen , D. Z. Li , and T. S. Yi . 2017. “Plastomes of Mimosoideae: Structural and Size Variation, Sequence Divergence, and Phylogenetic Implication.” Tree Genetics & Genomes 13: 41.

[ece374416-bib-0066] Wu, C. S. , C. I. Chen , and S. M. Chaw . 2022. “Plastid Phylogenomics and Plastome Evolution in the Morning Glory Family (Convolvulaceae).” Frontiers in Plant Science 13: 1061174. 10.3389/fpls.2022.1061174 PMC9808526 36605953

[ece374416-bib-0067] Wu, H. , D. Z. Li , and P. F. Ma . 2024. “Unprecedented Variation Pattern of Plastid Genomes and the Potential Role in Adaptive Evolution in Poales.” BMC Biology 22: 97. 10.1186/s12915-024-01890-5 PMC11057118 38679718

[ece374416-bib-0068] Wu, Z. Y. , J. Liu , J. Provan , et al. 2018. “Testing Darwin's Transoceanic Dispersal Hypothesis for the Inland Nettle Family (Urticaceae).” Ecology Letters 21: 1515–1529. 10.1111/ele.13132 30133154

[ece374416-bib-0069] Wu, Z. Y. , R. I. Milne , J. Liu , et al. 2022. “Phylogenomics and Evolutionary History of Oreocnide (Urticaceae) Shed Light on Recent Geological and Climatic Events in SE Asia.” Molecular Phylogenetics and Evolution 175: 107555. 10.1016/j.ympev.2022.107555 35724818

[ece374416-bib-0070] Wu, Z. Y. , A. K. Monro , R. I. Milne , et al. 2013. “Molecular Phylogeny of the Nettle Family (Urticaceae) Inferred From Multiple Loci of Three Genomes and Extensive Generic Sampling.” Molecular Phylogenetics and Evolution 69: 814–827. 10.1016/j.ympev.2013.06.022 23850510

[ece374416-bib-0071] Zhan, M. , L. Xue , J. J. Zhou , et al. 2024. “Polyphyly of Boehmeria (Urticaceae) Congruent With Plastome Structural Variation.” Frontiers in Plant Science 15: 1297499. 10.3389/fpls.2024.1297499 PMC11319286 39139721

