# Supplementary material for: Plastid Phylogenomics of Elatostemateae (Urticaceae): Insights Into Structural Variation and Phylogenetic Relationships
Source: Ecol Evol. 2026 Oct 6;16(10):e74416. doi: 10.1002/ece3.74416 (PMC13641976; doi:10.1002/ece3.74416)
Supplement: Supplementary file 1 [file ECE3-16-e74416-s002.docx]

Table S1. Substitution models used for different datasets and tree reconstruction methods.

| Dataset | Best substitution model for ML tree | Best substitution model for BI tree |
| --- | --- | --- |
| CDS | GTR+F+I+I+R5 | GTR+I+G |
| CP genome | TVM+F+R5 | GTR+I+G |
| nrDNA | GTR+F+I+I+R3 | GTR+I+G |
| CP genome + nrDNA | GTR+F+I+I+R4 | GTR+I+G |
